# Objects Versus Shadows as Influences on Perceived Object Motion

**DOI:** 10.1177/2041669516677843

**Published:** 2016-11-22

**Authors:** Marouane Ouhnana, Frederick A. A. Kingdom

**Affiliations:** Department of Ophthalmology, McGill Vision Research, Montreal, QC, Canada

**Keywords:** 3D perception, higher order motion, perceptual organization, spatial cognition

## Abstract

The motion trajectory of an object’s cast shadow has been shown to alter the perceived trajectory of a casting object, an effect that holds even if the cast shadow appears unrealistic. This raises the question of whether a cast shadow per se is necessary for this influence, a question that has been studied only with stationary targets. We examined the relative influence of a shadow and a spherical object on the perceived motion trajectory of an identical spherical object, using a paradigm similar to Kersten, Mamassian, and Knill's ball-in-box animation. We recorded both depth and height estimates of the perceived end-point of the target trajectory as a function of various target and context trajectories. Both shadows and objects significantly influenced the perceived trajectory of the target, though the influence of the shadow was overall stronger. We conjecture that the influence of the object reveals the assumption that similar objects moving at the same speed and in similar directions are perceived to move within the same plane, a plane subject to a fronto-parallel bias.

## Introduction

A single retinal projection may correspond to a multitude of perceptual interpretations, yet our visual experience is seldom ambiguous. Contextual cues in our visual environment tend to disambiguate an otherwise ambiguous retinal input. Shadows are such a cue; they have been shown to have a profound impact on the perceived position and motion trajectory of the casting object to which they are perceived to be attached (Kersten, Knill, Mamassian, & Bülthoff, 1996; Kersten, Mamassian, & Knill, 1994; Ni, Braunstein, & Andersen, 2004).

When one talks about shadows, one may refer to either attached or cast shadows: the former refers to a region of an object receiving weaker illumination from a light source, whereas the latter refers to a region of a surface or object that is occluded from the light source (see review by Mamassian, Knill, & Kersten, 1998). In this communication, we are interested in cast shadows, and how their influences compare to those of a secondary object on the perceived motion direction of a target object.

The first exploration of this question was undertaken by Ni et al. (2004) using a display comprised of two flat disks, one above the other, set against a grass-textured ground plane. The lower disc had various thicknesses and was textured to either resemble the upper disc or shaded dark to resemble a shadow. The discs were either stationary or moved horizontally back and forth along with the background to simulate head movements, but without motion parallax. The display therefore simulated a stationary object in both situations. The task was to estimate the perceived distance of the upper disc. Results showed that the upper disc was perceived to be significantly closer to the ground-contact position of the lower disk when the latter appeared as a shadow compared to when it was shaded to resemble the upper disc.

Cast shadows are tied to the objects that cast them, with the exact position of a shadow also determined by the position of the light source. Thus, when an object moves, so does its cast shadow. Hence, the influence of a shadow is likely to be greatest when both the casting object and the shadow are in motion. Kersten et al. (1994) demonstrated that the motion trajectory of a cast shadow significantly altered the perceived motion trajectory of the casting object—a spherical ball. When the shadow moved diagonally, the casting object appeared to recede and move along the ground, whereas when it moved horizontally, the casting object appeared to rise up in the frontal plane. Interestingly, the influence of the shadow was found to occur even when its contrast polarity was reversed. An interesting corollary to the flexibility with which shadows exert their influence is that incongruent shadows, that is, those whose positions and shapes do not accord with the laws of physical optics, are nevertheless perceived as shadows (Casati, 2008; Cavanagh, 2005; Mamassian, 2008), albeit with some measurable perceptual costs (Castiello, 2001). Such perceptual flexibility raises the possibility that Gestalt principles alone might be sufficient to explain the perceptual shifts observed in Kersten et al.’s (1994) moving ball experiments. The Gestalt principle that springs to mind is common-fate, which states that objects that appear to move together group together (Wertheimer, 1923). Common-fate may have a corollary: Objects that group together are perceived to move together. Such a grouping principle might even predict a stronger influence of a similar object on the target than a shadow. On the other hand, shadows may be a more powerful contextual cue due to the tight constraints under which they occur in the natural world.

The aim of this communication is to examine the relative influence of shadows and objects on the perceived motion trajectory of a primary object. For this purpose, we have employed a paradigm similar to Kersten et al.’s (1994) ball-in-box-animation, with observers making perceptual estimates of the motion trajectory of a dynamic target object in the presence of diverging trajectories of either cast shadows or a secondary bottom object.

## General Methods

### Subjects

Five observers participated in the experiment. All observers were naïve to the experimental aims and had normal or corrected-to-normal visual acuity. Written consent was obtained from all test participants, and all experimental protocols were approved by the McGill University Research Ethics Board.

### Apparatus

The stimuli were created using open-source 3D computer graphics software Blender version 2.67 b. The stimuli were displayed using MATLAB version 8.1 with the Psychophysics Toolbox Version 3 extensions. The stimuli were presented on an Apple LED Cinema display connected to an Apple Mac Pro. The resolution of the display was set to 2,560 × 1,440 with a refresh rate of 60 Hz. Observers were seated 60 cm away from the display.

### Stimuli

**Figure 1.**
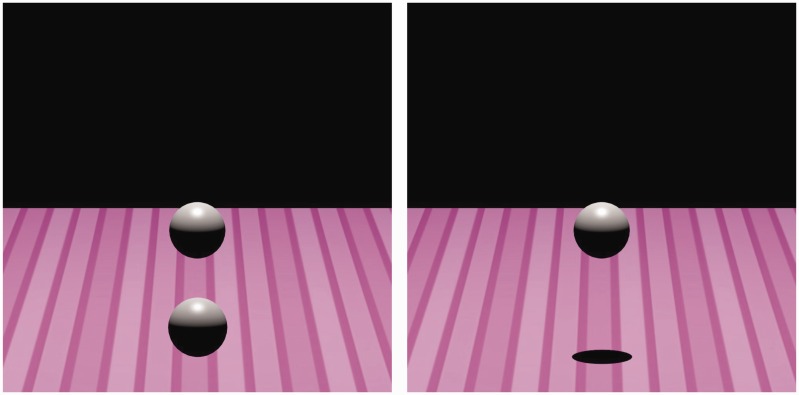
Target (top-sphere) and two context conditions: left, an identical secondary sphere; right, a cast shadow.

**Figure 2.**
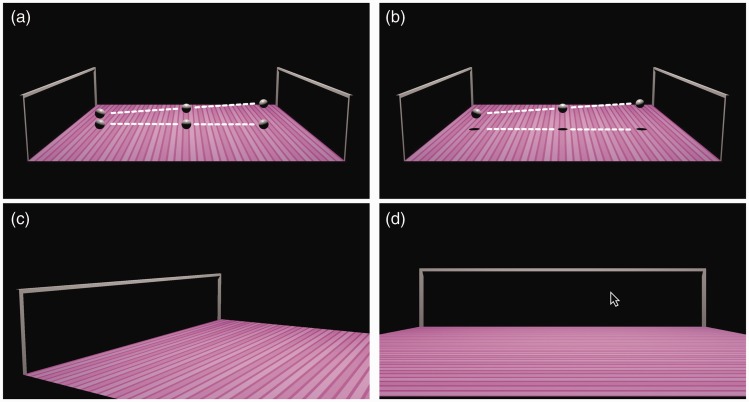
Procedure for measuring the perceived trajectories of the target, for secondary sphere (a) and cast shadow (b) contexts. The white dashed lines show the trajectories (from left to right). Following the animation, the target and context were removed and the display rotated (c) to present the skeleton goal posts fin planar view (d), at which point a cursor appeared. The observer’s task was to indicate via mouse-click the location where they thought the target would cross the goal, termed the “end-point” of the perceived trajectory.

**Figure 3.**
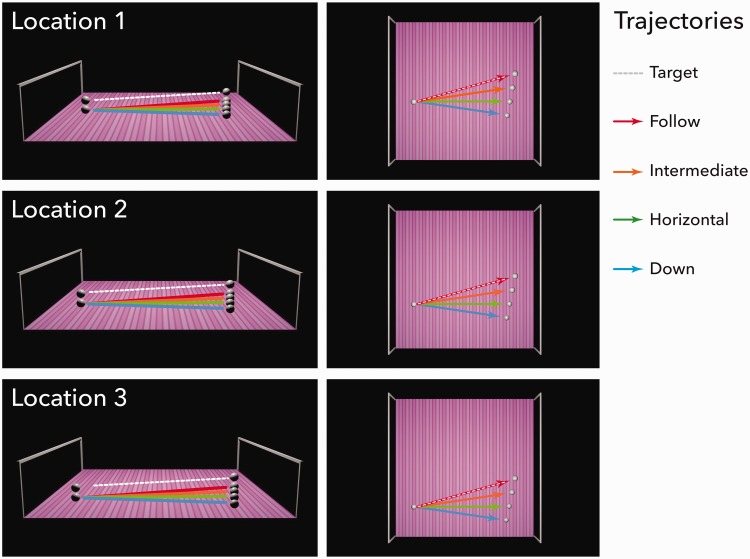
Starting locations and context trajectories. The figures on the left illustrate the three different starting locations of the target and context figures. The trajectory of the target sphere is shown by the dashed line and is fixed for each starting location. The four trajectories of the context figure (here the sphere) are the continuous lines, and their names are shown on the right. The figures on the right show a bird’s eye view of the trajectories. Horizontally flipped versions of all trajectories were also employed but are not illustrated here.

A display similar to the (Kersten et al., 1994) ball-in-box-animation was recreated using Blender. A spherical light source simulated lighting from above and placed in XYZ Blender space (0, 0, 20). The energy and distance of the light source were set to 6 and 100, respectively. The camera was placed in XYZ Blender space of (0, –34, 8) with a XYZ rotation of (80, 0, 0) using perspective view with a focal length of 35. Rendered animations had a resolution of 1,980 × 1,280 and each lasted 2 s. Illustrations of the stimuli are shown in Figures 1 to 3.

### Target Stimulus

The target stimulus was created using Blender’s UV sphere mesh, shaded gray with specularities set to 0.5. The UV sphere mesh size was set to 0.41 for all XYZ Blender scale units. See the top sphere in both left and right hand side of Figure 1 for an example of the target sphere. The target sphere was rendered following a specific fixed diagonal trajectory for each of the starting location conditions and was described later in the text.

### Context Stimuli

Context stimuli were created in a similar manner to the target stimulus and positioned below the target. The UV sphere mesh size was set to 0.37 for all XYZ Blender scale units. The size difference between the top and bottom UV sphere was designed to compensate for the camera’s perspective scaling, in other words to equate the perceptual size of both target and context stimuli. Two types of context figure were used, a cast shadow and a bottom-sphere identical to the target figure. When the context was a sphere, the shading was similar to the target stimulus. When the context was a cast shadow, the UV sphere was set to 100% transparency and set to cast a shadow. Figure 1 shows both context stimuli with the target. The context stimuli could follow four possible trajectories each starting at one of three possible locations, and these are described later.

### Ground Surface

A textured surface was rendered with an XYZ Blender scale of (10, 10, 01) and placed in the center of the 3D space. Rectangular skeleton “goal posts” were rendered on each side of the textured surface and represented the region within which observers could position their responses, as shown in Figure 2. The goal posts were rendered with an XYZ Blender scale of (0.1, 0.1, 2.1) with the crossbar defined by an XYZ Blender scale of (0.1, 10, 0.1) units.

### Procedure

Observers were first presented with an animation of the target and context figure, or without the context during baseline trials, moving across the display. Then, the target, and context if applicable, disappeared, and the display was rotated by changing the camera’s perspective to present the goal posts in frontal view; see Figure 2 for an example trial. Observers were instructed to indicate via a mouse-click the location where they thought the target would cross the goal.

Observers were presented with one of three possible target sphere start and end locations. The start locations are described by the following XYZ Blender positions. See Figure 3 for an illustration of each of the possible starting locations.

Location 1: starting location (–7.2, –1.5, 1.33), ending location (7.2, 2.5, 1.45)

Location 2: start location (–7.2, –3.5, 1.33), ending location (7.2, 0.5, 1.45)

Location 3: start location (–7.2, –5.5, 1.33), ending location (7.2, –1.5, 1.45)

Regardless of the start or end location of the target sphere, its motion followed a linear trajectory oriented at 4° from horizontal.

Each target-sphere animation was accompanied by one of four context (secondary sphere or cast shadow) trajectories, as well as a no-context baseline trajectory. For all context trajectories, the XY Blender start locations were identical to those of the target-sphere, with Z Blender space set to a value 0.41, and the context’s X Blender location adjusted to be perceptually below the target. The following context trajectories, illustrated in Figure 3, were tested:

*Follow*: The Y Blender position was the same as the top-sphere and followed the same linear trajectory oriented 4° from horizontal.
  *Intermediate*: A value of 2 units was subtracted from the Y Blender ending position of the top-sphere. This motion trajectory followed a linear path orientated 2° from horizontal.
  *Horizontal:* A value of 4 units was subtracted from the Y Blender ending position of the top-sphere. This motion trajectory followed a horizontal path, that is, 0° from horizontal.
  *Down*: A value of 6 units was subtracted from the Y Blender ending position of the top-sphere. This motion trajectory followed a linear path oriented –2° from horizontal.

Horizontally flipped versions of all conditions were also presented. All conditions, horizontally flipped and nonflipped, were interleaved and tested 10 times.

## Results

**Figure 4.**
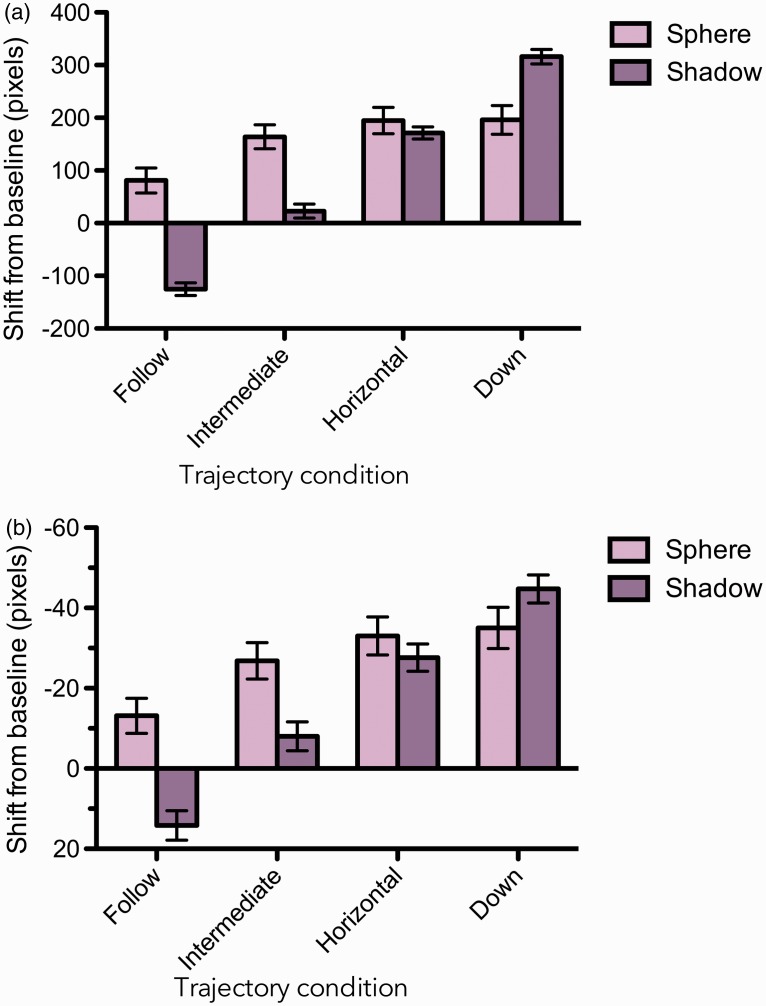
Shift in end-point from baseline for the sphere and shadow contexts in terms of (a) depth and (b) height. The error bars correspond to 95% confidence intervals calculated across trials.

To assess the influence of the context trajectories on the perceived trajectories of the target, end point estimates were separated into depths (X) and heights (Y). To normalize the data across experimental conditions, the observer’s baseline depth and height estimate for each starting location and for both flipped and non-flipped animations was subtracted from the corresponding trajectory estimates. We term these measures *shifts from baseline*. The normalized depth and height estimates were then averaged for each condition. The shifts from baseline averaged across all subjects’ trial data are summarized in Figure 4 for both cast shadow and secondary sphere context conditions and for each context trajectory.

To analyze the influence of context on the perceived target trajectories, two separate two-factor (Context × Trajectory) within-subject ANOVAS (analyses of variance) were conducted, one on the depth estimates the other on the height estimates. There was a significant main effect of trajectory for both depth, *F*(3, 12) = 37.06, *p* < .0001, and height, *F*(3, 12) = 16.61, *p* < .001, estimates, showing that the contexts had a significant impact on the perceived trajectories of the target. There was no significant difference between the two contexts (shadow and bottom-sphere) for either depth, *F*(1, 12) = 0.65, *p* < .46, or height, *F*(1, 12) = 1.30, *p* < .32, estimates. However, this lack of difference between the two contexts obscures differences in the *pattern* of perceived target trajectories, which can be seen in Figure 4 and is expressed in the significant interaction between context and trajectory, for both depth, *F*(3, 12) = 32.38, *p* < .0001, and height, *F*(3, 12) = 13.94, *p* < .001, estimates. As the figure shows, the form of this interaction is that the depth and height estimates of the target followed more closely the changes in trajectory for the cast shadow compared with those of the secondary sphere contexts.

To investigate whether the shifts in target trajectory estimates occurred for *both* the cast shadow and secondary sphere contexts, a simple effects test was conducted between trajectories for each context condition. The analysis revealed significant effects of context trajectory for both context conditions (for the shadow condition, depth: *F*(3, 20) = 9.15, *p* < .01; height: *F*(3, 20) = 5.33, *p* < .05. For the secondary sphere condition, depth: *F*(3, 20) = 113.7, *p* < .0001; height: *F*(3,20) = 48.95, *p* < .0001.

To assess whether each change in context trajectory was accompanied by a change in target trajectory estimate, we conducted Tukey’s Honestly Significant Difference (HSD) pairwise comparison tests between the various pairs of trajectories. For the cast shadow context, Tukey’s HSD test revealed that all trajectories had a significant differential influence on target estimates. In other words as the trajectory of the cast shadow deviated away from the target, there was an accompanying significant shift in depth estimates: all *p*’s < .01. This coherent relationship between cast shadow trajectory and target estimates was also found for height (*p*’s < .01), with the exception of the follow with intermediate trajectory comparison (*p* = 1), and the horizontal with down trajectory comparison (*p* = .23). In the case of the secondary sphere context condition, Tukey’s HSD test revealed a significant shift in depth estimates between the horizontal and follow context trajectories (*p* < .05) as well as between the down and follow context trajectories (*p* < .05). All other secondary sphere trajectory comparisons were not significantly different from each other, that is, produced similar shifts from baseline in both depth and height estimates.

**Figure 5.**
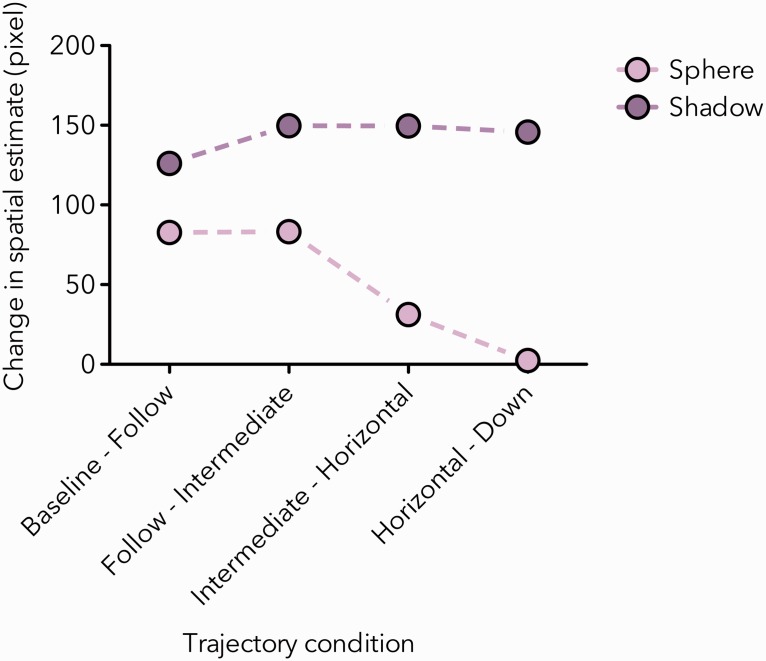
Euclidean distances between end-point estimates for adjacent pairs of context trajectories for both secondary sphere and cast shadow contexts.

To further illustrate the relationship between target end-point estimates and context, the Euclidean distances between target trajectory end-point estimates for each adjacent pair of context trajectories were calculated and are summarized in Figure 5. The figure reveals consistent shifts in estimates for the cast shadow, while estimates for the secondary sphere display asymptote beginning at the second context trajectory.

## Discussion

Our goal was to compare the influence of a secondary sphere and the cast shadow on the perceived motion trajectory of a target sphere. The main findings are as follows:

1. The perceived motion trajectory of a moving target was found to be significantly influenced by the motion trajectory of both a cast shadow and secondary sphere context.
2. The perceived target motion trajectory diverged along with the divergence of the cast shadow motion trajectory, but reached a plateau in the secondary sphere condition at greater motion trajectory divergences.

Our data from the shadow conditions replicate the findings of Kersten et al. (1994) and add the new finding that there is a similar influence of an identical object. The influence of the object however is overall less than that of the shadow, in keeping with the results of Ni et al. (2004), who compared shadow and object influences on the perceived location of a stationary object. The question however remains: Why does the secondary object have any influence at all, and why is the influence less than that of a shadow?

One idea suggested by an anonymous reviewer is motion repulsion. The angle between two superimposed dot patterns moving in different motion directions tends to be perceptually overestimated (Rauber & Treue, 1999), an example of a more general effect termed *acute-angle expansion* (e.g., Kitaoka & Ishihara, 2000). The largest overestimation occurs at a motion angle of about 7°, and diminishes in magnitude for both smaller and larger angles (Rauber & Treue, 1999). In our study, the trajectory angles ranged between 0° and 6°. Therefore, there may have been a perceived overestimation in the trajectory divergences in our displays. However, the task for the subject was not to estimate the angular difference in trajectory, but to estimate the height and depth of the perceived end-point of the target trajectory. The effect of motion repulsion would, if anything, tend to shift the end-point of the target away from the trajectory of the sphere, whereas our results showed the opposite. Thus, while we cannot rule out an influence of motion repulsion on our results, it seems highly unlikely that it is their main cause.

Another possibility is that the context sphere, which is identical to the target sphere, is perceived as its mirror-reflection, as if the ground plane in the display acted like a mirror. The physics of reflection is compatible with any of the trajectory combinations of perceived target motion direction and context motion direction used in the study, though normally of course the shading on the reflected sphere would be above the sphere, not below it as in our display. Mirror-reflection is therefore a tantalizing possibility.

The Gestalt principle of common fate states that objects that appear to move together group together. The target and secondary sphere share a degree of common fate in that they exhibit similar motion speeds, not too dissimilar motion directions, and a common vertical axis in their 2D projections. Grouping might lead to the interpretation that the two objects are perceived to lie on the same, flat, rigid surface. For the conditions in our experiment, any surface formed from such object grouping would be increasingly slanted away from the observer as the trajectory of the secondary sphere diverged from that of the target toward the observer. However, if there was a bias toward perceiving that surface as close to fronto-parallel, similar to the bias observed in the stereopsis domain (Deas & Wilcox, 2014; Fahle & Westheimer, 1988; Liu, David, & Ronen, 1999), then the target object would tend to appear to move upwards, as we found. This reduction in perceived surface slant would occur in conjunction with the trajectory of the secondary sphere, which would define the tilt of the surface.

As to why the cast shadow exerts a bigger influence than the secondary object, the answer probably lies in the fact that shadows are more constrained in the natural world than other objects in terms of their physical relationships with the objects that cast them. Kersten et al. (1994) suggest that the visual system assumes a stationary light source when dealing with shadows. Coupled with the fact that light is typically assumed to come from above (Morgenstern, Murray, & Harris, 2011; Ramachandran, 1988) and that shadows are assumed to lie on the ground plane, this reduces significantly the possible interpretations of the target-shadow trajectories in Kersten et al.’s (1994) and our moving ball experiments.

## Conclusion

The perceived motion direction of an object such as a sphere is influenced by the motion direction of an identical object, not just a cast shadow. Future research will be needed to determine the underlying cause of this influence, exploring such possibilities as mirror-reflection and a bias towards motion surfaces being fronto-parallel. In conclusion, we have demonstrated systematic shifts of end point estimates to trajectory divergences of a secondary object.
